# Prospective Evidence on Artificial Intelligence−Assisted Melanoma Diagnostics: A Systematic Review and Meta-Analysis

**DOI:** 10.1001/jamadermatol.2026.0217

**Published:** 2026-03-25

**Authors:** Sara Laiouar-Pedari, Arlene Kühn, Christoph Wies, Carina Nogueira Garcia, Jana Therés Winterstein, Lukas Heinlein, Annemarie Hoffsommer, Tirtha Chanda, Sarah Haggenmüller, Titus J. Brinker

**Affiliations:** 1 Division of Digital Prevention, Diagnostics and Therapy Guidance, German Cancer Research Center, Heidelberg, Germany; 2 Medical Faculty, University Heidelberg, Heidelberg, Germany

## Abstract

**Importance:**

Dermoscopy is a standard of care for melanoma diagnostics, and artificial intelligence (AI) systems are increasingly investigated as decision-support tools. Prospective evidence is essential to assess their performance compared to dermatologists.

**Objectives:**

To evaluate the diagnostic performance of dermatologists, AI systems, and dermatologists assisted by AI in prospective studies of melanoma detection, and to assess the readiness of AI for clinical use.

**Data Sources:**

PubMed, Embase, Web of Science, and Google Scholar were searched from inception through July 9, 2025.

**Study Selection:**

Eligible studies were prospective, used dermoscopic images, and reported or allowed calculation of performance metrics for dermatologists, AI, or dermatologists assisted by AI against a histopathologic reference standard. Nondermoscopic comparators and retrospective designs were excluded. Studies with 20 or fewer histopathologically confirmed melanomas were excluded a priori from quantitative synthesis.

**Data Extraction and Synthesis:**

Two reviewers independently screened and extracted data and discrepancies or missing values were clarified among all authors. Risk of bias and applicability were assessed with QUADAS-2 and QUADAS-C. Study-level sensitivity and specificity were summarized and plotted; head-to-head comparisons were analyzed descriptively.

**Main Outcomes and Measures:**

Diagnostic outcomes were sensitivity, specificity, accuracy, and balanced accuracy for melanoma detection.

**Results:**

Eleven prospective studies with a total of more than 2500 patients and 50 participant-dermatologists were included in the analyses. Dermatologists achieved a pooled sensitivity of 78.6% (95% CI, 67.5%-88.1%) and specificity of 75.2% (95% CI, 63.3%-84.3%), whereas AI alone reached 80.9% (95% CI, 63.6%-94.5%) sensitivity and 75.6% (95% CI, 64.5%-85.6%) specificity. In the single study reporting AI-assisted dermatologists, sensitivity was 91.9% and specificity was 83.7%. In direct clinical comparisons, AI demonstrated higher specificity and similar sensitivity. Most studies were at high risk of bias in patient selection and index test domains, primarily due to the preselection of lesions suspected of melanoma and binary classifications.

**Conclusions and Relevance:**

In the systematic review and meta-analysis of prospective settings, AI systems perform at comparable levels to dermatologists for melanoma diagnostics and may enhance performance when used as a decision-support tool. However, the frequent risk of bias and limited generalizability of current studies highlight the need for broader validation in unselected patient populations in the clinical setting.

## Key Points

**Question:**

How does the diagnostic performance of artificial intelligence (AI) for melanoma in prospective dermoscopy studies compare with that of dermatologists?

**Findings:**

Across 11 prospective studies including more than 2500 participants, AI and dermatologists showed comparable diagnostic performance. However, the evidence base remains small, and study designs are heterogeneous, with a high risk of bias in patient selection and index test domains.

**Meaning:**

Although current findings support the potential clinical application of AI, validation remains at an early stage because larger, multicenter, and methodologically rigorous prospective studies are required to confirm the safety and clinical utility of AI in routine practice.

## Teaser

This systematic review and meta-analysis evaluates the diagnostic performance of dermatologists with and without artificial intelligence support in studies of melanoma detection.

## Introduction

Malignant melanoma is among the most aggressive forms of skin cancer. Early detection and timely intervention are crucial for improving patient outcomes.^1^ Dermoscopy, a noninvasive diagnostic technique, has become an indispensable tool in dermatology by enabling improved visualization of subsurface skin structures. Compared to unaided visual inspection, it substantially increases diagnostic accuracy for melanoma^1^ and helps to reduce unnecessary excisions of benign lesions.^1,2,3,4,5^ Despite being the current standard of care, however, its diagnostic performance remains highly dependent on the clinician’s level of experience.^6^

In recent years, artificial intelligence (AI) systems have shown promising results in the automated analysis and classification of dermoscopic images. Convolutional neural networks (CNNs), in particular, have become the most commonly used approach in this field and form the basis for most modern AI models for classifying skin lesions.^7,8^ Numerous retrospective studies have reported diagnostic performances comparable to, or even exceeding, those of expert dermatologists.^9,10,11^ These findings have fueled growing interest in the integration of AI as decision-support tools to enhance melanoma detection in clinical practice.

Nevertheless, most studies evaluating AI performance have been retrospective and rely on curated image datasets that may not reflect the complexity of everyday clinical practice. Retrospective analyses are limited in their ability to assess generalizability, risk of bias, and true diagnostic impact. To address this gap, we conducted a meta-analysis focusing exclusively on prospective studies that compared the diagnostic performance of dermoscopy alone with dermoscopy supported by AI, or AI alone, in melanoma detection. By synthesizing prospective evidence, this systematic review and meta-analysis aims to inform the current state of clinical readiness for AI in melanoma diagnostics and to identify key areas requiring further validation.

## Methods

This systematical review and meta-analysis used only previously published data and was therefore exempted from review by the Ethics Committee of the Faculty of Medicine, Heidelberg University. The study was conducted in accordance with the Preferred Reporting Items for Systematic Reviews and Meta-Analyses (PRISMA) reporting guidelines^12^ and the protocol was registered with PROSPERO on July 21, 2025 (CRD420251084932).^13^

### Study Eligibility Criteria

The Population/Patient/Problem, Intervention, Comparison, and Outcome (PICO) framework^14^ guided the definition of eligibility. Included studies assessed populations of adult patients (age ≥18 years) with skin lesions suspected of malignant melanoma; interventions involving the application of AI to dermoscopic images; compared diagnostic assessments by dermatologists using dermoscopy, with or without AI support; and their primary outcomes were diagnostic performance, expressed through sensitivity, specificity, accuracy, and/or balanced accuracy. Beyond the criteria defined by the PICO framework, studies were required to be peer-reviewed, be reported in English, and have a prospective design. The latter was defined as the collection and assessment of in vivo data for both dermatologist and AI evaluations. To ensure statistical robustness, included studies also had to enroll at least 20 histopathologically confirmed melanoma cases. Studies with only 1 relevant study group (eg, AI only or dermatologist only) were also eligible because the meta-analysis allowed for indirect comparisons across modalities. Interventional studies were only included if a diagnostic end point was reported.

A study was excluded if it reported combined malignant diseases without separate data for melanoma, described algorithm development without clinical validation (eg, on open databases or challenge datasets), or originated from computer science contexts without clinical application. Within the eligible studies, we excluded any study groups that did not meet our criteria, including retrospective groups and those using clinical images instead of dermoscopic images or that used nondermoscopic comparators.

### Search Strategy

A systematic literature review was conducted of PubMed, Google Scholar, Embase, and Web of Science for studies published between January 1, 2000, and July 9, 2025. The search strategy combined terms related to melanoma, dermoscopy, AI, and prospective design. Full search strategies for each database are provided in eTables 1 to 3 in Supplement 1.

### Reference Management and Study Selection

The reference management tool, Zotero,^15^ was used to manage citations and remove duplicates. Title and abstract screening were performed independently by 2 reviewers (S.L.P. and A.K.). Potentially eligible full-text articles were retrieved and independently assessed for inclusion by the same reviewers.

Based on the predefined search criteria, a total of 308 publications were identified across the 4 databases. After removing duplicates and articles that were not peer-reviewed, 176 records remained and were independently screened. Disagreements were resolved through discussion. Reasons for excluding other prospective studies are provided in eTable 4 in Supplement 1.

### Data Extraction and Synthesis

Two reviewers (S.L.P. and A.K.) independently extracted metric data and key features for each study. Eligible studies reported performance metrics for melanoma diagnostics validated against histopathologic test results. When metrics were not reported, they were calculated from available raw data. Using recorded true positives (TP), true negatives (TN), false positives (FP), and false negatives (FN), the following measures were derived: sensitivity = TP/(TP + FN); specificity = TN/(FP + TN); accuracy = (TP + TN)/(TP + FN + FP + TN); and balanced accuracy = (sensitivity + specificity)/2.

In cases of inconsistencies or missing values, the study authors were contacted for clarification. If no response was received within 1 week, missing values were recalculated from the raw data (eMethods in Supplement 1).

Performance metrics were systematically extracted and categorized by diagnostic approach: dermatologists alone, AI alone, or dermatologists assisted by AI. A meta-analysis was subsequently performed to pool performance estimates for each diagnostic modality, based on data from all included studies.

### Critical Appraisal

Risk of bias was evaluated using the Quality Assessment of Diagnostic Accuracy Studies 2 (QUADAS-2) tool,^16^ and, when appropriate, the QUADAS-Comparative (QUADAS-C) tool,^17^ specifically designed for comparative diagnostic accuracy studies. Two reviewers (S.L.P. and A.K.) conducted the assessment independently, and any disagreements were resolved through consultation with a third reviewer (C.N.G.). The overall quality of studies was evaluated by considering the proportion of studies with high vs low risk of bias, and the consistency of reported performance metrics across studies.

### Statistical Analysis

If not directly reported, sensitivity, specificity, accuracy, and balanced accuracy were calculated from 2 × 2 contingency tables provided in the studies. If 95% CIs for sensitivity or specificity were not reported, they were estimated using the bootstrap method; the same approach was applied to derive pooled-matrix CIs at study level.^18^ For method-specific sensitivity and specificity, pooled metrics were calculated by averaging all metrics within a given study group. The summary receiver operating characteristic (SROC) curve was derived according to the approach proposed by Moses et al.^19^

Statistical analyses were performed using R, version 4.1.2 (R Foundation for Statistical Computing) using the boot package, version 1.3-28, and the base stats package. Forest plots were generated with the forest plot package, version 3.1.1, and SROC and the box plots were created using the ggplot2 package, version 2.4.4. Differences between groups were evaluated using the nonparametric Wilcoxon rank sum test.

## Results

**Figure 1.**
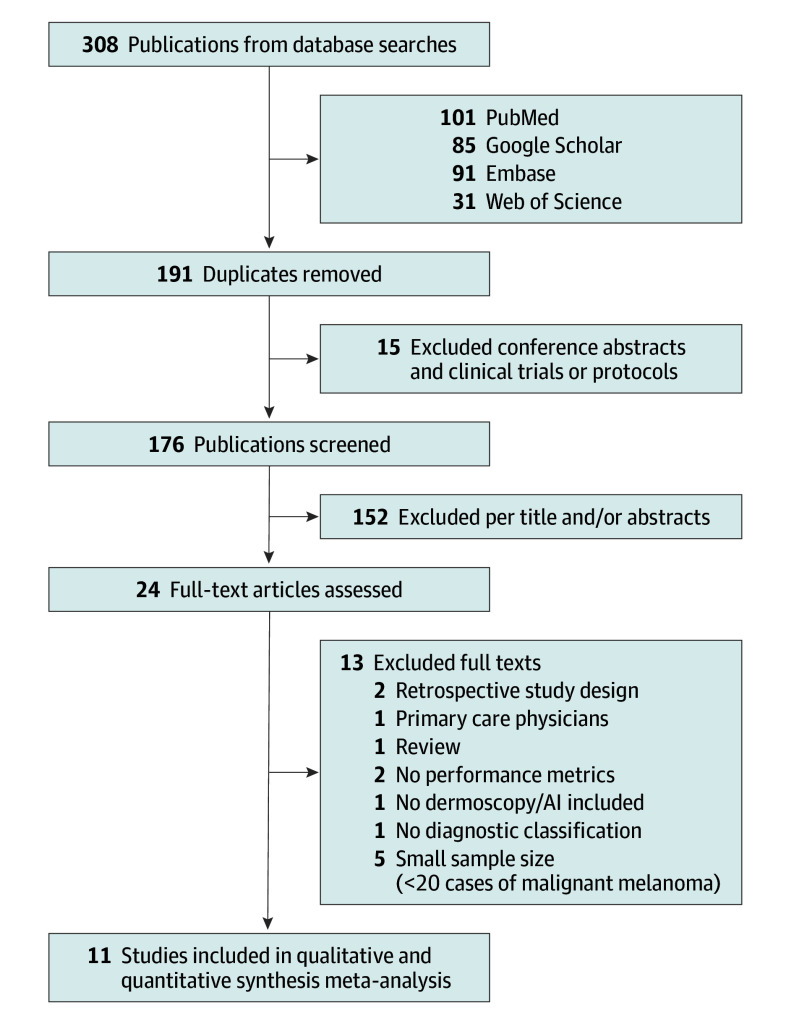
PRISMA Flow Diagram of Included Studies AI indicates artificial intelligence.

Eleven studies met the predefined eligibility criteria (Figure 1), evaluating the diagnostic performance of dermatologists alone, AI alone, or dermatologists assisted by AI in prospective dermoscopic-image settings. Findings of histopathologic testing of melanoma-suspected lesions served as the reference standard in all studies. For benign lesions, the reference standard varied across studies and included histopathologic results, clinical follow-up, or expert consensus. Study populations differed considerably, with malignant melanoma cases ranging from 26 to 653 and nonmalignant cases, from 88 to 4495. Eight studies directly compared dermatologists’ performance with that of AI.

Among the 11 eligible studies, 3 study groups were excluded due to retrospective design^20,21^ or the use of clinical rather than dermoscopic images.^22^ Additionally, some of the studies used comparators outside the scope of this meta-analysis, such as general physicians using AI,^23^ confocal laser scanning microscopy,^24^ naked-eye examination, and telespectrophotometry,^25^ teledermatology, spectroscopy, or multispectral imaging^26^; therefore, these nondermoscopic study groups were excluded from the analysis. Furthermore, 1 study assessing dermatologist performance with AI support reported only area under the curve (AUC) without sensitivity or specificity metrics and thus, could not be included in the meta-analysis.^27^

In total, 9 studies reported performance metrics for dermatologists, 5 for AI alone, and 1 for dermatologists supported by AI (the Table). All included AI studies used CNN-based methods. Thomas et al^28^ reported 4 distinct AI performance outcomes—it was conducted across 2 clinical sites and evaluated 2 versions of the CNN algorithm (ie, DERM-vA and DERM-vB) following an update during the study period, and was designed as a reader study with a prospective AI group. Maclellan et al^26^ and Menzies et al^29^ each reported results for 2 different AI algorithms. In addition, Menzies et al^29^ stratified dermatologist performance by level of experience (ie, expert vs novice; both trained in the evaluation of pigmented skin lesions). Given that the remaining studies did not stratify by experience, all dermatologist groups were pooled into 1 single group. Menzies et al^29^ further noted that AI assessments were available within approximately 1 hour, which was considered sufficiently prospective for inclusion in our systematic review and meta-analysis.

**Table.** Study Characteristics of Included Studies^a^

Source	Cases/patients	Ground truth	Study group	Diagnostic categories or classification methods or reason for exclusion
Phillips et al,^20^ 2019	MM (n = 125), others (n = 426)/(n = 514)	HPE for suspected lesions	Dermatologists group	MM, dysplastic nevi, or other lesions
			Not included: SkinAnalytics (AI algorithm)	Data for AI classification were prospectively collected but retrospectively analyzed
Heinlein et al,^21^ 2024	MM (n = 653), others (n = 918)/(n = 1716)	HPE for all lesions	Dermatologists group	MM vs non-MM (dysplastic nevi or other lesions)
			Not included: “all data are external” (ADAE) algorithm (CNN models)	Data for AI classification were prospectively collected but retrospectively analyzed
Maier et al,^22^ 2015	MM (n = 26), others (n = 119)/NA	HPE for all lesions	Dermatologists group (n = 2)	MM vs non-MM (dysplastic nevi + nevi)
			Not included: SkinVision app (CNN model)	Clinical images used for AI analysis
Dreiseitl et al,^23^ 2009	MM (n = 27), others (n = 431)/(n = 511)	HPE for suspected lesions; follow-up for other lesions	Dermatologists group (n = 1)	MM vs non-MM
			Not included: nonexpert physicians + MoleMax II	Nonexpert physicians
Langley et al,^24^ 2007	MM (n = 37) others (n = 88)/(n = 125)	HPE for all lesions	Dermatologists group (n = 1)	Melanocytic nevi vs MM
			Not included: Confocal scanning laser microscopy	No dermoscopic or AI system
Bono et al,^25^ 2002	MM (n = 66), others (n = 247)/(n = 298)	HPE for all lesions	Dermatologists group (n = 1)	MM vs non-MM
			Not included: naked-eye telespectrophotometry	Naked eye and telespectrosphotometry are not dermoscopic techniques
MacLellan et al,^26^ 2021	MM (n = 59), others (n = 150)/(n = 184)	HPE for all lesions	Dermatologists group (n = 2)	Management decision instead of diagnosis: excise, do not excise, observe
			Not included: teledermatologists, MelaFind, Verisante Aura	Teledermatology and for AI, no dermoscopic systems included
			AI group: FotoFinder Pro (CNN model)	Probability score between 0 and 1, with a threshold for melanoma of at least 0.5
			AI group: FotoFinder Tueb (CNN model)	Probability score between 0 and 1, with a threshold for melanoma of at least 0.5
Marchetti et al,^27^ 2023	MM (n = 95), others (n = 508)/(n = 435)	HPE for suspected lesions	AI group: AI algorithm (ADAE-CNN models)	Probability malignancy score
			Not included: dermatologists (n = 11) + ADAE (CNN model)	Only AUC metrics
Thomas et al,^28^ 2023	MM (n = 140), others (n = 4635)/NA	HPE for suspected lesions; CA or HPE for other lesions	AI group: DERM vA (CNN model) clinic 1	7-Class classification: MM, SCC, BCC, IEC, AK, AN, or benign
	MM (n = 58), others (n = 2527)/NA		AI group: DERM vB (CNN model) clinic 1	7-Class classification: MM, SCC, BCC, IEC, AK, AN, or benign
	MM (n = 33), others (n = 676)/NA		AI group: DERM vA (CNN model) clinic 2	7-Class classification: MM, SCC, BCC, IEC, AK, AN, or benign
	MM (n = 18), others (n = 624)/NA		AI group: DERM vB (CNN model) clinic 2	7-Class classification: MM, SCC, BCC, IEC, AK, AN, or benign
Menzies et al,^29^ 2023	MM (n = 55), others (n = 117)/(n = 124)	HPE for suspected lesions	Dermatologists group (n = 5)	Single best diagnosis out of 7-class classification: MM, MN, BCC, pAK/IEC, BKL, BVL, and DF
			Dermatologists group: novice (n = 18)	
			AI group: MetaOptima; 7-class (CNN models)	The maximum probability class is returned as a prediction for the 7-class diagnostic algorithm
			AI group: MetaOptima (CNN models)	7-Class classification (results obtained within 1 h)
Winkler et al,^30^ 2023	MM (n = 38), other (n = 190)/(n = 188)	HPE for suspected lesions; follow-up and/or EC for other lesions	Dermatologists group (n = 22)	Malignancy score between 0 and 1, with a threshold for malignancy of at least 0.5
			AI group: Fotofinder Pro (CNN model)	Malignancy score between 0 and 1, with a threshold for malignancy of at least 0.5
			Dermatologist + AI group: dermatologists + FotoFinder Pro (CNN model)	Malignancy score between 0 and 1, with a threshold for malignancy of at least 0.5

Abbreviations: ADAE, an open-source CNN-based melanoma detection algorithm; AI, artificial intelligence; AN, atypical nevus; AUC, area under the curve; BCC, basal cell carcinoma; BKL, benign keratotic lesion; BVL, benign vascular lesion; CA, clinical assessment; CNN, convolutional neural network; DF, dermatofibroma; EC, expert consensus; FotoFinder Pro, FotoFinder Moleanalyzer Pro; FotoFinder Tueb, FotoFinder Moleanalyzer Tuebinger; HPE, histopathologic examination; IEC, intraepithelial carcinoma; MM, malignant melanoma; MN, melanocytic nevus; NA, not applicable; pAK, pigmented actinic keratosis; vA, version A; vB, version B.

^a^ Overview of key features of all studies meeting the inclusion criteria: 9 studies included dermatologist-only groups (dermatologist group), 5 AI-only groups (AI group), and 1 AI-assisted dermatologist group (dermatologist + AI group). Some study groups did not meet the eligibility criteria for specific setups and, therefore, were excluded from the quantitative synthesis (labeled as not included).

### Quality of Studies

**Figure 2.**
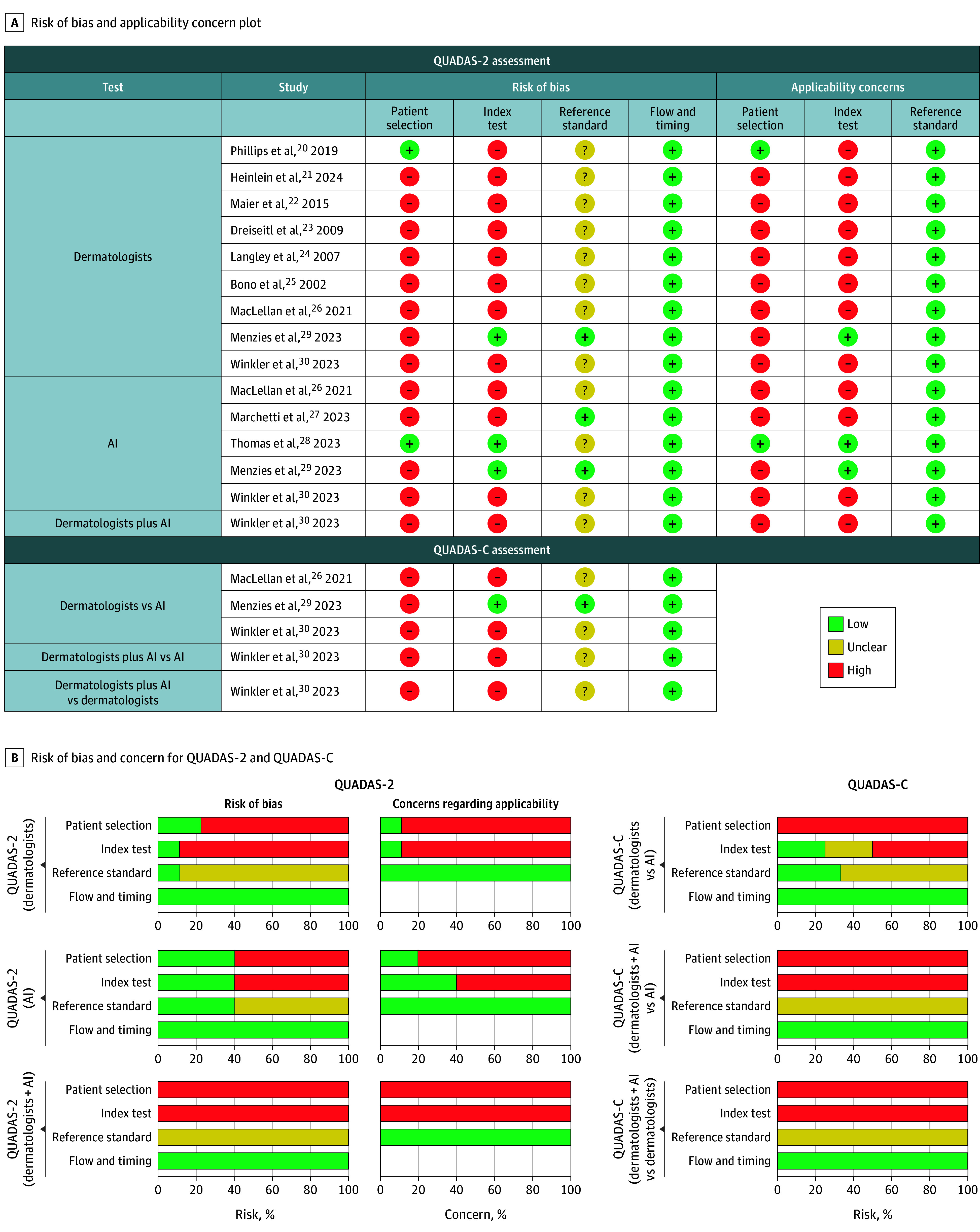
Heat Map and Bar Charts of QUADAS-2 and QUADAS-C Risk of Bias and Applicability Concerns Across All Eligible Studies (n = 11) A, Risk of bias and applicability concern plot shows individual judgments across each QUADAS-2 and QUADAS-C domains. Boxes indicate risk of bias or concern. B, Bar charts show the proportion of studies rated at low, unclear, or high risk or concern for each QUADAS-2 and QUADAS-C domain. Study groups were assessed separately: dermatologists (n = 9), AI (n = 5), and dermatologists + AI (n = 1). For comparative designs (n = 3), QUADAS-C domains were applied: dermatologists vs AI (n = 3), dermatologists supported by vs AI (n = 1), and dermatologists supported by AI vs dermatologists (n = 1). AI indicates artificial intelligence; QUADAS-2, Quality Assessment of Diagnostic Accuracy Studies 2 tool; QUADAS-C, the Quality Assessment of Diagnostic Accuracy Studies Comparative tool.

We assessed the 11 eligible studies using the QUADAS-2 tool (Figure 2). Moreover, the QUADAS-C extension was applied in 3 studies, comparing diagnostic approaches (dermatologists vs AI).^26,29,30^ In the study by Winkler et al,^30^ a pairwise comparison was conducted among 3 groups (dermatologists, AI, and dermatologists plus AI), as the risk of bias and applicability domains differed between groups, and therefore, needed to be evaluated separately (Figure 2).

All 11 studies were conducted prospectively and enrolled consecutive patients in clinical practice. However, in 9 studies, patients were preselected, with inclusion restricted to melanoma-suspected, melanocytic, or pigmented lesions.^21,22,23,24,25,26,27,29,30^ This design introduced patient selection bias given that many benign lesions typically encountered in routine practice were excluded. This limitation also contributed to applicability concerns in patient selection because in actual clinical settings, all patients would be considered.

In the index test domain, 1 study^23^ adopted a patientwise rather than a lesionwise classification of melanoma. This approach inflates diagnostic accuracy because a patient is counted as correctly diagnosed if any melanoma lesion is identified, even if other lesions are misclassified.

Another study^26^ defined the diagnostic outcome based on the dermatologists’ management decision. Although all lesions were excised for verification, thus avoiding verification bias, this design introduced an artificial reduction in specificity given that many benign lesions not judged for excision were nonetheless removed and counted as false positives.

Nine studies applied a binary classification (malignant melanoma vs nonmalignant melanoma) rather than a multiclass approach (Table). This introduces index test bias because pooling all nonmalignant melanoma conditions can obscure differences in diagnostic performance across specific differential diagnoses, potentially over- or underestimating sensitivity and specificity. Moreover, the binary setup was deemed to have high applicability concerns because it does not reflect actual clinical practice, during which dermatologists must distinguish malignant melanoma from a variety of benign and malignant lesions. In contrast, studies using multiclass classifications^28,29^ more closely reflected clinical decision-making and therefore were considered to have a lower risk of bias.

In 9 studies, it was not reported whether pathologists had prior knowledge of the dermatologists’ diagnoses when conducting the histopathologic assessments.^20,21,22,23,24,25,26,28,30^ This lack of information was classified as unclear risk of bias in the reference standard domain. However, this does not constitute an applicability concern because in routine clinical practice, pathologists typically have access to the dermatologists’ clinical assessment (Figure 2).

### Diagnostic Performance

The malignant melanoma diagnostic performance of the 11 eligible studies was assessed using sensitivity, specificity, accuracy, and balanced accuracy, either as reported or calculated from published data (eTable 2 in Supplement 1). Dermatologists’ performance was reported in 9 studies, AI performance in 5 studies, and dermatologists’ performance assisted by AI in 1 study.

In 1 study,^20^ the sample size in each cohort appears to have been interchanged, leading us to recalculate the results with new values^22^ (eTable 9 in Supplement 1 provides the details). Another study^26^ provided specificity metrics that did not align with the reported diagnostic contingency table; therefore, we recalculated its specificity (eTable 12 in Supplement 1 provides the details). When further inconsistencies could not be resolved by contacting the original authors, we applied our predefined procedure of recalculating values based on available raw data.

**Figure 3.**
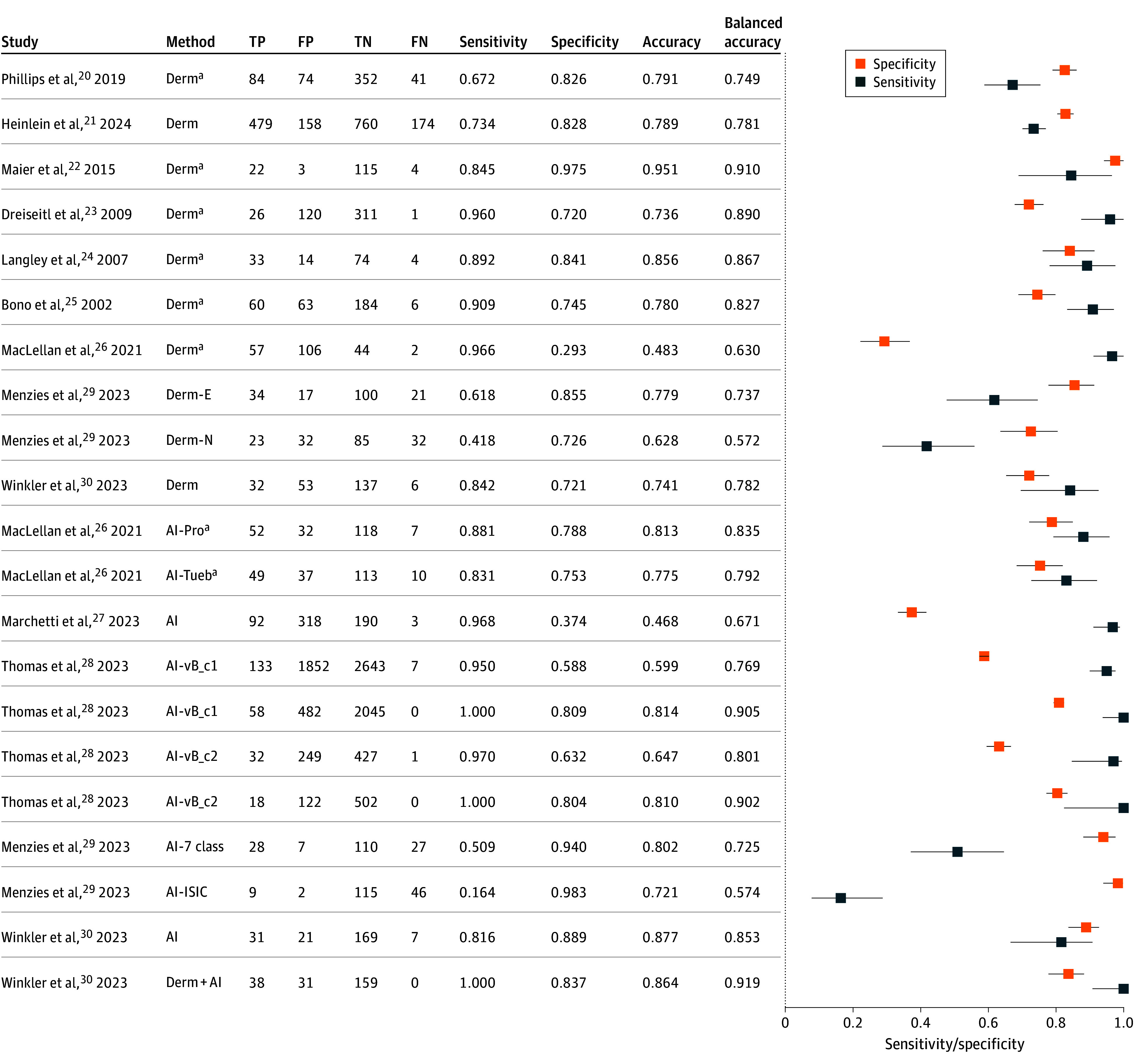
Forest Plots of the Sensitivity and Specificity of the Eligible Studies Sensitivity, specificity, accuracy, and balanced accuracy were reported for each study. The results were stratified into dermatologists’ performance, AI performance, and dermatologists’ performance supported by AI. AI indicates artificial intelligence; AI-Pro, FotoFinder Pro; AI-Tueb, FotoFinder Tuebinger; AI-vA_c1, AI version A clinic 1; AI-vB_c1, AI version B clinic 1; AI-vA_c2, AI version A clinic 2; AI-vB_c2, AI version B clinic 2; Derm-E, expert dermatologists; Derm-N, novice dermatologists; FN, false negative; FP, false positive; ISIC, International Skin Imaging Collaboration; TN, true negative; TP, true positive. ^a^Estimated 95% CI were used if they were not reported or were unreliable.

**Figure 4.**
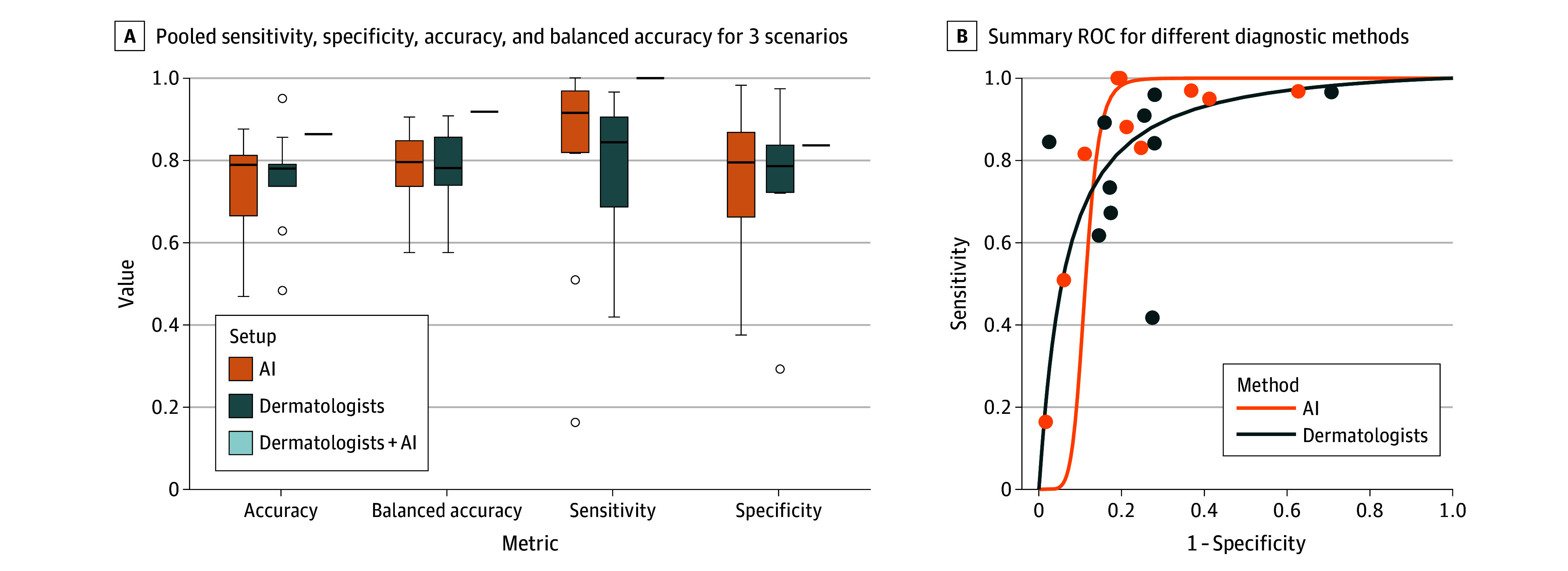
Box Plots and Summary Receiver Operating Characteristic (ROC) Curves of the Eligible Studies A, Box plots of all diagnostic metrics across all 3 setups, illustrating pooled sensitivity, specificity, accuracy, and balanced accuracy for dermatologists, AI, and dermatologists supported by AI. The horizontal line indicates the median; the upper and lower box limits denote the first and third quartiles. The ends extend to 1.5 times the IQR. The dots represent outliers that are beyond the third quartile. Dermatologists (n = 10), AI (n = 10), and dermatologists supported by AI (black line; n = 1). Outliers are represented as points. B, SROC for different diagnostic methods curves for different diagnostic methods. Study-level sensitivity and specificity estimates are shown, stratified by dermatologists and AI. AI indicates artificial intelligence.

Across the 9 studies reporting dermatologists’ performance, sensitivity ranged from 41.8% to 96.6%, and specificity from 29.3% to 97.0% (Figure 3). The pooled estimates across all 10 investigators were 78.6% (95% CI, 67.5%-88.1%) sensitivity, 75.3% (95% CI, 63.3%-84.3%) specificity, 75.3% (95% CI, 67.6%-82.3%) accuracy, and 77.4% (95% CI, 70.8%-83.6%) balanced accuracy (Figure 4A; eTable 5 in Supplement 1).

For the 5 studies reporting AI performance, sensitivity ranged from 16.4% to 100.0%, and specificity, from 37.4% to 98.3% (Figure 3), with pooled estimates across all 10 investigators of 80.9% (95% CI, 63.6%-94.5%) sensitivity, 75.6% (95% CI, 64.5%-85.6%) specificity, 73.3% (95% CI, 65.4%-80.0%) accuracy, and 78.3% (95% CI, 72.0%-84.1%) balanced accuracy (Figure 4A; eTable 5 in Supplement 1). The single study reporting dermatologists’ performance assisted by AI achieved a sensitivity of 91.9% and a specificity of 83.7%, corresponding to 86.4% accuracy and 87.8% balanced accuracy (eTable 5 in Supplement 1; Figure 3). Figure 4B shows the SROC curves with the trade-off between sensitivity and specificity across studies, providing a comprehensive summary of overall diagnostic performance. Although neither the statistical analysis (eTable 5 in Supplement 1) nor Figure 4 indicate significant differences between dermatologists and AI, the point estimates of the aggregated metrics uniformly favored AI.

Across the 3 prospective head-to-head studies,^26,29,30^ AI systems consistently demonstrated higher specificity but similar or lower sensitivity compared with dermatologists (Figure 3; eTable 6 in Supplement 1 provides the head-to-head value comparisons).

## Discussion

This systematic review and meta-analysis assessed prospective studies comparing dermatologists, AI alone, and AI-assisted dermatologists to evaluate diagnostic performance and clinical readiness. Prospective evidence indicates that AI performs at a level comparable to dermatologists in melanoma diagnosis, with similar pooled sensitivity (80.9% vs 78.6%), specificity (75.6% vs 75.3%), accuracy (73.3% vs 75.3%), and balanced accuracy (78.3% vs 77.4%) even under actual clinical conditions, suggesting its potential as a decision-support tool beyond retrospective benchmarks, which often overestimate performance.^10^ Notably, models trained on larger and more diverse databases performed better in clinical practice,^29^ highlighting the importance of dataset size and heterogeneity for real-world applicability.

Although pooled estimates suggest similar overall performance between AI and dermatologists, direct head-to-head comparisons within the same clinical setting^26,29,30^ indicate higher specificity (94.0% and 98.3% vs 85.5%^29^; 78.8% and 75.3% vs 29.3%^26^; 88.9% vs 72.1%^30^) at comparable sensitivity for AI (50.9% vs 61.8%^29^; 88.1% and 83.1% vs 96.6%^26^; 81.6% vs 84.2%^30^). This observation may be explained by the fact that dermatologists tend to act cautiously and are more likely to recommend biopsy in cases of diagnostic uncertainty.^31,32^ In contrast, AI-based assessments could help reduce unnecessary biopsies.

Compared with previous evidence syntheses, notable differences emerge. The pooled sensitivity (78.6%) and specificity (75.2%) for dermatologists in our meta-analysis (studies published between 2002 and 2024) were lower than the estimates reported by Vestergaard et al^3^ in 2008 (sensitivity 0.87 and specificity 0.91, based on studies from 1993-2006). Notably, this meta-analysis compared dermoscopy findings with unaided clinical examination in studies conducted mostly in specialist referral clinics and often limited to lesions suspected of melanoma, with several using rule-based dermoscopic criteria, design features that may partly explain the higher reported accuracy. Earlier studies^33,34,35,36,37,38,39,40^ often included more clinically evident melanomas and straightforward benign lesions, inflating accuracy via spectrum effects, a phenomenon where the performance of a diagnostic test varies across different patient populations. In contrast, more recent studies^21,29^ tend to include diagnostically challenging lesion sets, use multicenter designs, and, in some cases, run head-to-head with AI systems—design choices that enrich equivocal cases. Taken together, our findings represent a more realistic reflection of contemporary dermatologists’ performance across diverse clinical settings.

### Limitations

While these findings highlight the potential clinical value of AI-assisted diagnosis, they must be interpreted with caution, considering several methodologic limitations. Notably, patient selection introduces a systematic bias: in 9 of the 11 included studies, participants were preselected to include only melanoma-suspected, melanocytic, or pigmented lesions. This does not reflect the broader spectrum of lesions encountered in daily clinical practice and likely results in overestimated sensitivity (fewer FNs) and underestimated specificity (given that benign routine lesions are underrepresented). Consequently, the reported diagnostic performance may not fully generalize to routine settings.

Other design-related biases also influence interpretation. Dreiseitl et al^23^ calculated diagnostic accuracy on a patientwise rather than lesionwise basis, which inflates sensitivity because identifying a single melanoma lesion in a patient counts as a correct diagnosis, even if additional lesions are misclassified. In the study by MacLellan et al,^26^ the dermatologist’s management recommendation (excise, not excise, or watch) was treated as a melanoma classification. For verification, however, all lesions were excised, so many benign lesions initially considered for excision were counted as FPs. This design eliminated verification bias but led to an artificial reduction in specificity.

Similarly, several studies used binary classification (melanoma vs nonmelanoma), which oversimplifies the diagnostic task and may obscure differences in distinguishing between specific benign and malignant conditions. These design types reduce applicability given that clinical practice requires differentiation across multiple lesion types.

Another important limitation of the included studies is the imbalance in evidence: 9 studies reported dermatologist performance, 5 assessed AI alone, and only 1 investigated dermatologist with AI support. This makes it questionable to draw meaningful conclusions regarding the added value of AI support in routine care. In addition, most AI studies are still early in their prospective validation, and performance may be vulnerable to domain shifts between training and clinical application. Factors such as image quality, device heterogeneity, and patient selection can lead to deviations from retrospective performance benchmarks. Our inclusion of prospective designs addresses some of these concerns, but the evidence base remains limited.

## Conclusions

This systematic review and meta-analysis found prospective evidence indicating that AI achieves dermatologist-level performance for dermoscopic melanoma diagnosis, with no significant differences in pooled sensitivity or specificity. This finding is encouraging for clinical translation. Yet, the diversity of study designs, risks of bias, and the limited number of high-quality prospective datasets highlight that AI is still in the early phase of clinical validation. Larger, multicenter, and methodologically rigorous prospective studies with unselected, real-world patient populations will be essential to determine the reliability, safety, and added value of AI in routine clinical practice.

## Notes

Supplement 1. eTable 1. Search categories and search terms according to the PICO model eTable 2. Final Search Strings in PubMed, Embase, and Web of Science (WoS) and Search Results eTable 3. Final Search Strings in Google Scholar and Search Results (July, 09 2025) eTable 4. Exclusion of studies and reasoning eTable 5. Pooled diagnostic performance, expressed as sensitivity, specificity, accuracy, and balanced accuracy eTable 6. Sensitivity and Specificity values of head-to-head studies comparing dermatologists and AI performances eTable 7. Values for the study of Phillips et al eTable 8. Values for the study of Heinlein et al eTable 9. Values for the study of Maier et al., 2015 eTable 10: Values for the study of Dreiseitl et al., 2009 eTable 11. Values for the study of Langley et al., 2007 eTable 12. Values for the study of MacLellan et al., 2021 eTable 13. Values for the study of Marchetti et al., 2023 eTable 14. Values for the study of Thomas et al. 2023

Supplement 2. Data Sharing Statement
