# Supplementary material for: Prospective Evidence on Artificial Intelligence−Assisted Melanoma Diagnostics: A Systematic Review and Meta-Analysis
Source: JAMA Dermatol. 2026 Mar 25;162(5):478–87. doi: 10.1001/jamadermatol.2026.0217 (PMC13019344; doi:10.1001/jamadermatol.2026.0217)
Supplement: Supplement 1. — eTable 1. Search categories and search terms according to the PICO model eTable 2. Final Search Strings in PubMed, Embase, and Web of Science (WoS) and Search Results eTable 3. Final Search Strings in Google Scholar and Search Results (July, 09 2025) eTable 4. Exclusion of studies and reasoning eTable 5. Pooled diagnostic performance, expressed as sensitivity, specificity, accuracy, and balanced accuracy eTable 6. Sensitivity and Specificity values of head-to-head studies comparing dermatologists and AI performances eTable 7. Values for the study of Phillips et al eTable 8. Values for the study of Heinlein et al eTable 9. Values for the study of Maier et al., 2015 eTable 10: Values for the study of Dreiseitl et al., 2009 eTable 11. Values for the study of Langley et al., 2007 eTable 12. Values for the study of MacLellan et al., 2021 eTable 13. Values for the study of Marchetti et al., 2023 eTable 14. Values for the study of Thomas et al. 2023 [file jamadermatol-e260217-s001.pdf]

Supplemental Online Content

Laiouar-Pedari S, Kühn A, Wies C, et al. Prospective evidence on augmented intelligence−assisted melanoma diagnostics: a systematic review and meta-analysis. *JAMA Dermatol.* Published online March 25, 2026. doi:10.1001/jamadermatol.2026.0217

eTable 1. Search categories and search terms according to the PICO model

eTable 2. Final Search Strings in PubMed, Embase, and Web of Science (WoS) and Search Results

eTable 3. Final Search Strings in Google Scholar and Search Results (July, 09 2025)

eTable 4. Exclusion of studies and reasoning

eTable 5. Pooled diagnostic performance, expressed as sensitivity, specificity, accuracy, and balanced accuracy

eTable 6. Sensitivity and Specificity values of head-to-head studies comparing dermatologists and AI performances

eTable 7. Values for the study of Phillips et al

eTable 8. Values for the study of Heinlein et al

eTable 9. Values for the study of Maier et al., 2015

eTable 10 : Values for the study of Dreiseitl et al., 2009

eTable 11. Values for the study of Langley et al., 2007

eTable 12. Values for the study of MacLellan et al., 2021

eTable 13. Values for the study of Marchetti et al., 2023

eTable 14. Values for the study of Thomas et al. 2023

This supplemental material has been provided by the authors to give readers additional information about their work.

© 2026 Brinker TJ et al. *JAMA Dermatol.*

eTable 1: Search categories and search terms according to the PICO model

Search Category	Search Terms (Keywords)
**Patient, Population, Conditions** (Indication): Adults with melanoma suspicious skin lesions	Melanoma, skin cancer
**Intervention** (medical Purpose): Prospective dermoscopic image analysis of melanoma suspicious skin lesions using AI-based decision support systems	AI, artificial intelligence, machine learning, deep learning, convolutional neural network, ML, DL, CNN, prospective, dermoscopic
**Comparison**: Prospective analysis of melanoma suspicious skin lesions using dermatoscopy by a dermatologist	Physician, doctor, dermatologist, melanoma screening, skin examination, visual inspection, prospective, dermatoscope, dermatoscopy
**Outcome:** Diagnosis of malignant melanoma	Diagnosis of melanoma, diagnosis

The databases for the literature search were selected according to the recommendations of the Harvard Countway Library (https://guides.library.harvard.edu/meta-analysis/databases) and included PubMed, Embase, Web of Science, and Google Scholar. For PubMed, Embase, and Web of Science, identical search strings could be applied. In contrast, the search strategy for Google Scholar had to be adapted, as the original search string used in the other databases yielded an unmanageable number of results (>20,000).

© 2026 Brinker TJ et al. *JAMA Dermatol*.

eTable 2: Final Search Strings in **PubMed**, Embase, and Web of Science (WoS) and Search Results

Final search string (combination of categories)	***Filter Title/Abstract***	***PubMed 09.07.25***	***Embase 07.07.25***	***WoS 09.07.25***
(melanoma OR "skin cancer") AND diagnosis AND (dermatoscopy OR dermatoscope OR dermoscopic) AND ((skin OR melanoma OR visual) AND (examination OR inspection OR screening)) AND (physician OR doctor OR dermatologist) AND prospective	*Title/Abstract*	*67*	*42*	*15*
(melanoma OR "skin cancer") AND diagnosis AND (" artificial intelligence" OR AI OR "machine learning" OR ML OR " deep learning" OR DL OR "convolutional neural network" OR CNN) AND (dermoscopic OR dermatoscope OR dermatoscopy) AND prospective	*Title/Abstract*	*34*	*49*	*16*
	Search results:	*101*	*91*	*31*
	Duplicates:	*25*	*11*	*3*
	Duplicates with PubMed:	*-*	*31*	*23*
	Duplicates with Google Scholar:	*15*	*2*	*1*
	Results without Duplicates:	*61*	*47*	*4*

eTable 3: Final Search Strings in Google Scholar and Search Results (July, 09 2025)

Final search string (combination of categories)	***Filter Title/Abstract***	***Search results***
diagnosis AND dermatoscope AND intitle: "melanoma" AND (" artificial intelligence" OR AI OR "machine learning" OR ML OR "deep learning" OR DL OR "convolutional neural network" OR CNN) AND intitle: "prospective"		*10*
diagnosis AND dermatoscopy AND ((skin OR melanoma OR visual) AND (examination OR inspection OR screening)) AND (physician OR doctor OR dermatologist) AND intitle: melanoma AND intitle: prospective		*74*
	Search results:	*84*
	Duplicates:	*6*
	Results without Duplicates:	*78*

© 2026 Brinker TJ et al. *JAMA Dermatol*.

eTable 4: Exclusion of studies and reasoning

Study	***Cases***	***Method***	***Reasons for Exclusion***
Papachristou et al., 2024 [33]	*MM n=25, Others n=232*	*Dermalyser*	*primary care physicians, not dermatologists*
Marsden et al., 2024 [34]	*MM n=8, Others= 232*	*Derm, SkinAnalytics*	*malignant vs. non-malignant classification, teledermatology, few melanomas*
Jahn et al, 2022 [35]	*MM n=6, Others = n=55*	*SkinVision, Dermatologist + AI*	*few melanomas*
Bono, 2002 [36]	*MM n= 13, Others n=148*	*Dermatologists*	*few melanomas*
Perrinaud Gaide, 2007 [37]	*MM n=9, Others n=98*	*Dermatologists 3 different Systems*	*few melanomas, only a prospective data set with retrospective analysis*
Van der Rhee, 2010 [38]	*MM n=14, Others n=195*	*Dermatologists*	*few melanomas, focus on the management decision*
Van der Rhee, 2011 [39]	*MM n=2, Others= 47*	*Dermatologists*	*few melanomas, focus on families with a melanoma history and management decisions*
Durdu, 2011 [40]	*MM n=10, Others n=190*	*Dermatologists vs. Tzanck smear test*	*few melanomas*

© 2026 Brinker TJ et al. *JAMA Dermatol*.

eTable 5: Pooled diagnostic performance, expressed as sensitivity, specificity, accuracy, and balanced accuracy. Performance of dermatologists, AI, and dermatologists supported by AI for MM diagnostics. *N* indicates the number of investigators. The non-parametric unpaired Wilcoxon rank sum test was used for statistical analysis comparing dermatologists and AI; $p < 0.05$ was considered statistically significant.

Group	Sensitivity (95% CI)	Specificity (95% CI)	Accuracy (95% CI)	Balanced Accuracy (95% CI)
Dermatologists (n=10)	78.6% (67.5-88.1%)	75.3% (63.3-84.3%)	75.3% (67.6-82.3%)	77.4% 70.8-83.6%)
AI (n=10)	80.9% (63.6-94.5%)	75.6% (64.5-85.6%)	73.3% (65.4-80.0%)	78.3% (72.0-84.1%)
Dermatologists+AI (n=1)	91.9%	83.7%	86.4%	87.8%
p-values	0.34	0.97	1.0	0.86

eTable 6: Sensitivity and Specificity values of head-to-head studies comparing dermatologists and AI performances. Menzies et al. included an additional novice dermatologist group (Novice Derm) and evaluated two AI systems (AI-ISIC and AI-7-class). MacLellan et al. compared two different AI systems (FotoFinder Pro (AI-Pro) and FotoFinder Tübinger (AI-Tueb.)) to the dermatologist's arm. Sens=sensitivity, Spec=specificity.

Study	Dermatologists		Novice Derm.		AI performance		AI performance	
	Sens	Spec	Sens	Spec	Sens	Spec	Sens	Spec
Menzies [27]	61.8	85.5	41.8	72.6	AI-ISIC: 16.4	AI-ISIC: 98.3	AI-7-class: 50.9	AI-7-class: 94.0
Winkler [28]	84.2	72.1	-		81.6	88.9	-	
MacLellan [24]	96.6	29.3	-		AI- Pro: 88.1	AI- Pro: 78.8	AI- Tueb. 83.1	AI-Tueb. 75.3

© 2026 Brinker TJ et al. *JAMA Dermatol*.

eMethods. Calculations

Reported performance measures were extracted where available. When not provided, sensitivity (SEN), specificity (SPEC), accuracy (ACC), and balanced accuracy (BACC) were calculated from published true positive (TP), false positive (FP), true negatives (TN), and false negative (FN) values using standard formulas. Inconsistencies (e.g., implausible confidence intervals or mismatched lesion counts) were verified by contacting the study authors. If no response was received within one week, values were recalculated from available data. If the conventional equations were not used, the calculation method is given, with the values provided in the paper. Not reported and thus calculated values are noted in Supplementary eTables (*) and highlighted in the Results section.

Sensitivity = TP / (TP + FN)

Specificity = TN / (FP + TN)

Accuracy = (TP + TN) / (TP + FN + FP + TN)

Balanced Accuracy = (Sensitivity + Specificity) / 2

© 2026 Brinker TJ et al. *JAMA Dermatol*.

eTable 7: If not reported, SEN, SPEC, BACC, and ACC were calculated from reported or calculated TP, FN, FP, TN values for the study of Phillips et al., 2019 [20]. Calculated values are indicated with an asterisk (*). Sum of Melanoma cases correctly classified as “likely” or “highly likely” (TP); Sum of Melanoma cases classified as “unlikely” or “equivocal” (FN); Sum of Non-melanoma cases classified as “likely” or “highly likely” (FP); Sum Non-melanoma cases classified as “unlikely” or “equivocal” (TN).

**Investigator**	**TP***	**FN***	**FP***	**TN***	**SEN***	**SPEC***	**BACC***	**ACC***
**Dermatologists**	84 (36 + 48)	41 (9 + 32)	74 (8 + 6 + 35 + 25)	352 (80 + 35 + 155 + 82)	67.2%	82.6%	74,9%	79,1%

eTable 8: If not reported, SEN, SPEC, BACC, and ACC were calculated from reported or calculated TP, FN, FP, TN values for the study of Heinlein et al., 2024 [21]. Calculated values are indicated with an asterisk (*).

**Investigator**	**TP**	**FN***	**FP***	**TN***	**SEN***	**SPEC***	**BACC***	**ACC***
**Dermatologists**	479	174 (653 – 479)	158 (918 - 760)	760 (82.8% × 918)	73.4%	82.8%	78.1%	78.9%

© 2026 Brinker TJ et al. *JAMA Dermatol*.

eTable 9: If not reported**, SEN,** SPEC, BACC, and ACC were calculated from reported or calculated TP, FN, FP, TN values for the study of Maier et al., 2015 [22]. Calculated values are indicated with an asterisk (*). Maier et al calculated the dermatologists' sensitivity as 22 of 25 melanomas correctly classified, rather than 22 of 26 reported histologically verified melanomas. Likewise, specificity was reported as 115 of 119 correctly classified non-melanoma lesions, instead of 115 of 118 histologically verified benign lesions. We recalculated these values accordingly.

Investigator	TP	FN	FP	TN	SEN*	SPEC*	BACC*	ACC*
**Dermatologists**	22	4	3	115	84.6%	97.5%	91.0%	95.1%

eTable 10: If not reported, SEN, SPEC, BACC, and ACC were calculated from reported or calculated TP, FN, FP, TN values for the study of Dreiseitl et al., 2009 [23]**.** Calculated values are indicated with an asterisk (*).

Investigator	TP	FN	FP	TN	SEN	SPEC	BACC*	ACC*
**Dermatologists**	26	1	120	311	96.0%	72.0%	89.0%	73.6%

eTable 11: If not reported, SEN, SPEC, BACC, and ACC were calculated from reported or calculated TP, FN, FP, TN values for the study of Langley et al., 2007 [24]**.** Calculated values are indicated with an asterisk (*).

Investigator	TP	FN	FP	TN	SEN	SPEC	BACC*	ACC*
**Dermatologists**	33	4	14	74	89.2%	84.1%	86.7%*	85.6%*

© 2026 Brinker TJ et al. *JAMA Dermatol*.

eTable 12: If not reported, SEN, SPEC, BACC, and ACC were calculated from reported or calculated TP, FN, FP, TN values for the study of MacLellan et al., 2021 [26].The specificity reported by *MacLellan et al.* [24] did not align with our calculation. Based on the provided TN, TP, FN, and FP values, we recalculated the metric and derived a slightly different result. Calculated values are indicated with an asterisk (*).

Investigator	TP	FN	FP	TN	SEN*	SPEC*	BACC*	ACC*
**Dermatologists**	57	2	106	44	96.6%	29.3%	63.0%	48.3%

eTable 13: If **not** reported, SEN, SPEC, BACC, and ACC were calculated from reported or calculated TP, FN, FP, TN values for the study of Marchetti et al., 2023 [27]. Calculated values are indicated with an asterisk (*). The used AI algorithm is named ADAE.

Investigator	TP*	FN*	FP*	TN*	SEN	SPEC	BACC*	ACC*
**ADAE**	92 (96.8% x 95)	3 (95 - 92)	318 (603 - 95 - 190)	190 (37.4% × 508)	96.8%	37.4%	67.1%	46.8%

eTable 14: If not **reported**, SEN, SPEC, BACC, and ACC were calculated from reported or calculated TP, FN, FP, TN values for the study of Thomas et al. 2023 [28]. Calculated values are indicated with an asterisk (*). Thomas et al. reported four distinct AI performance outcomes, as the study was conducted across two clinical sites (clinic 1 and clinic 2) and evaluated two versions of the CNN algorithm (i.e., DERM-vA and DERM-vB) following an update during the study period.

Investigator	TP	FN	FP*	TN	SEN*	SPEC*	BACC*	ACC*
**DERM vA clinic 1**	133	7	1852 (4495 - 2643)	2643	95.0%	58.8%	76.9%	59.9%
**DERM vB clinic 1**	58	0	482 (2527 - 2045)	2045	100%	80.9%	90.5%	81.4%
**DERM vA clinic 2**	32	1	249 (676 - 427)	427	97.0%	63.2%	80.1%	64.7%
**DERM vB clinic 2**	18	0	122 (624 - 502)	502	100.0%	80.4%	90.2%	81.0%
