# Supplementary material for: Prospective Evidence on Artificial Intelligence−Assisted Melanoma Diagnostics: A Systematic Review and Meta-Analysis
Source: JAMA Dermatol. 2026 Mar 25;162(5):478–87. doi: 10.1001/jamadermatol.2026.0217 (PMC13019344; doi:10.1001/jamadermatol.2026.0217)
Supplement: Supplement 2. — Data Sharing Statement [file jamadermatol-e260217-s002.pdf]

Data Sharing Statement

Data

Data available: Yes
Data types: Data (not involving human participants)
How to access data: doi: 10.6084/m9.figshare.30346936
When available: With publication

Supporting Documents

Document types: None

Additional Information

Who can access the data: open access
Types of analyses: any purposes
Mechanisms of data availability: without investigator support
Any additional restrictions: no restriction
